# Vitamin D: A single initial dose is not bogus if followed by an appropriate maintenance intake

**DOI:** 10.1002/jbm4.10606

**Published:** 2022-02-18

**Authors:** Sunil J. Wimalawansa, Robin Whittle

**Affiliations:** ^1^ Department of Medicine, Cardio Metabolic Institute Somerset NJ USA; ^2^ Independent Researcher Daylesford Victoria Australia

## Peer Review

The peer review history for this article is available at https://publons.com/publon/10.1002/jbm4.10606.

**To the Editor:**

We read the review by Mazess and colleagues^(^ ^1^ ^)^ in *JBMR Plus* with interest. We have several points for comment and discussion.

First, a bolus dose is a single dose of a substance given over a short period, also called a loading dose.^(^ ^2^ ^)^ However, in the review,^(^ ^1^ ^)^ the term “bolus” dose refers to the nutrient cholecalciferol, vitamin D3 supplement as a longer‐term micronutrient, in larger quantities than accepted daily doses with intervals of over a month duration between intakes.

Second, although the authors rightly critique overly long intervals between vitamin D3 supplementation intakes (ie, >1‐month intervals), they were mistaken to refer to these as “bolus doses” and so mischaracterize bolus dosing as “bogus.”

In those with coronavirus disease(s) (e.g., COVID‐19), sepsis or other medical emergencies, achieving and mainteaning serum 25‐hydroxyvitamin D [25(OH)D] concentration of above 50 ng/mL is crucial for achieving proper immune function^(^ ^3^ ^)^ (also toreplete body stores) within days with a single bolus dose of vitamin D_3_, such as 5 to 10 mg (200,000 to 400,000 IU), or within 4 hours using a single, 0.5‐mg to 1‐mg oral dose of calcifediol (0.014 mg/kg body weight).^(^ ^4^ ^)^ However, to maintain the boosted serum 25(OH)D concentrations, D_3_ supplements should be taken daily or once a week in larger quantities (taking less frequently than this, is less effective).^(^ ^5^ ^)^

Third, the review's critique of the Murai study should not be considered a general critique of bolus D3 dosing. This trial resulted in poor clinical outcomes due to the faulty study design of using a bolus dose of D_3_ in seriously ill patients. Earlier administration of vitamon D_3_ and/or the use of rapidly acting calcifediol, would have been more effective in patients with advanced COVID‐19 and in sepsis.^(^ ^6^ ^)^

Finally, the circulatory 1,25‐dihydroxyvitamin D [1,25‐(OH)_2_D; calcitriol] levels and 24‐hydroxylase enzyme activity used in this review as indicators of immune system functions are not relevant to vitamin D status or to the critical, autocrine and paracrine signaling system of immune cells.

## Conflict of Interest

The authors declares no conflicts of interest.
